# Seasonal adjustment of the Spanish sales daily data

**DOI:** 10.1007/s13209-021-00251-7

**Published:** 2021-11-22

**Authors:** Ángel Cuevas, Ramiro Ledo, Enrique M. Quilis

**Affiliations:** Research and Statistics Division, Tax Agency, Spain

**Keywords:** Tax data, Daily time series, Short-term forecasts, Unobserved components model, C22, C32, C53

## Abstract

We present a procedure to perform seasonal adjustment over daily sales data. The model adjusts daily information from the Immediate Supply of Information System for Value Added Tax declaration forms compiled by the Spanish Tax Agency. The procedure performs signal extraction and forecasting at the daily frequency, by means of an unobserved components model. The daily information allows a permanently updated monitoring of the short-term economic conditions of the Spanish economy.

## Introduction

Economic data provided by tax sources are gaining popularity among economic analysts and forecasters due to its timely availability, reliability, coverage and direct economic meaning. In this way, tax-based data have a clear function in the design of a model aimed at nowcasting and short-term forecasting.

Updated and reliable forecasts play a critical role for budgetary planning and for the anticipation of risky situations due to adverse shocks. In particular, the daily information provided by the new Immediate Supply of Information system (SII, *Suministro Inmediato de Información*), based on the Value Added Tax (VAT) forms and developed by the Tax Agency, opens new perspectives for the integration, on a real-time basis, of tax-based reliable quantitative information with macro-data observed at lower frequencies.

This paper presents a system for seasonal adjustment of very high frequency data derived from tax sources. Specifically, the model treats the daily sales time series provided by the SII. Seasonal adjustment (SA) of daily data is necessary, in the same way as happens to monthly and quarterly data, in order to provide a meaningful signal of its underlying evolution. However, this task is notably more difficult than in the case of monthly or quarterly data due to the complexity of its seasonal component, formed by several subcomponents, some of them linked to fractional periodicities and its noisy nature (Ladiray et al. 2018).

The paper is organized as follows. The second section presents the main characteristic of the data. The third section develops the econometric methodology, which is implemented in two stages. In the first one, we apply a preliminary treatment of the deterministic effects and, in the second one, we use an univariate structural model, based on an unobserved component representation, to decompose and forecast the daily sales data. The empirical results are presented in the fourth section. The fifth section is focused in predictive evaluation. The paper ends presenting the main conclusions and future developments.

## Data

Daily sales series of monthly VAT taxpayers comes from the Immediate Supply of Information System (SII, *Sistema Inmediato de Información*), introduced in January 2017 and officially implemented by the Spanish Tax Agency since July 2017 (Tax Agency 2017).

This system allows the exchange of tax information between the Spanish Tax Agency and taxpayers required by the SII practically in real time, by supplying the detail of the invoicing records within four days, through the electronic platform of the Spanish Tax Agency.

In this way, both tax management and tax compliance are improved (e.g., by the taxpayers comparison of the information in their books with the information provided by their customers and suppliers).

The group compulsorily included in the SII is made up of all those taxpayers whose obligation to declare VAT is monthly:

- Large Companies (turnover greater than 6,010,214.04€ in the previous year).
- Those companies that pay taxes through the special regime for groups of companies.^1^
- Registered, voluntarily, in the Monthly VAT Return Registry.

In addition, this system is applied to those taxpayers who voluntarily adopt it.

Thus, the SII comprises about 63,000 taxpayers representing around 70% of the country’s total business turnover, with a great diversity of coverage by activities (see “Annex 1” for more details).

This statistic is particularly innovative at the international level both for its frequency (daily instead of monthly) and for its compilation procedure (exhaustive information based on administrative records instead of partial sampling surveys).

**Fig. 1:**
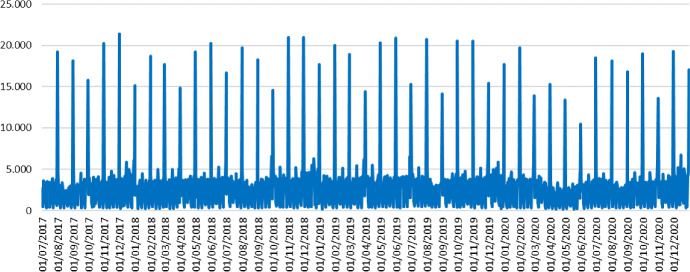
Daily sales from SII (million €)

Figure 1 shows the daily time series, from July 1st, 2017 to December 31th, 2020. The sample comprises from the full-fledged implementation of the SII until the end of 2020. At first glance, it is possible to see the large volatility of the series, due to the different invoicing patterns, that we will comment on later, such as the large effect of the end of the month, which makes very difficult to distinguish a relevant signal of underlying evolution.

As a general rule, firms must send their invoices four days after they have been issued. However, the information received by the Tax Agency is not always the final one, as it can be completed and corrected in the following days. Experience indicates that it is necessary to wait around two weeks for the stabilization of the levels that allows us to consider the data as definitive.

Moreover, it is appropriate to show how the daily information from the SII helps to anticipate the behavior of the domestic sales that are included in the monthly VAT data. Most of the companies that belonging to the SII must present the corresponding monthly periodic self-assessment forms. These companies can submit these self-assessment forms until the 25th of the month following the month to which they refer (except for the exceptions of December, which can be submitted until January 27, and January, which can be submitted until February 23).

**Fig. 2:**
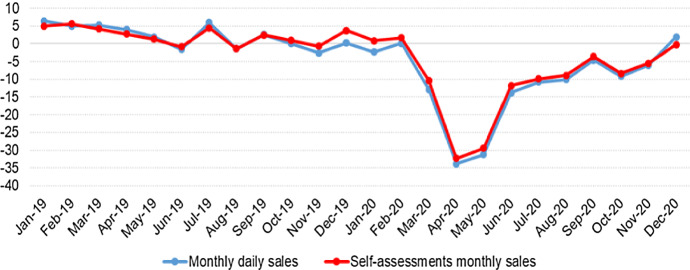
Daily sales versus self-assessments monthly sales, Y–o-Y rates

It should be noted that there are minor conceptual differences between the daily data and the data from self-assessment forms (mainly due to operations with reversed taxpayer, and their corresponding modifications). In order to determine whether these differences are very significant, Fig. 2 shows the evolution of the year-on-year growth rates of both sources for total domestic sales in 2019 and 2020.

We can appreciate the great similarity between both data sources, directionally and in magnitude (specifically, the correlation coefficient between both series is 0.99). So, the usefulness of the daily series is clearly demonstrated in order to anticipate its monthly counterpart.

The series are still at an experimental stage, requiring the approval by the Statistical Council in order to be included in the National Statistical Plan, in the same way as happened with the remaining statistics compiled by the Tax Agency (e.g., Sales, Employment and Wages in Tax Returns). Meanwhile, they are available for research purposes and upon request at directorestudiostributarios@correo.aeat.es.

## Econometric methods

In this section, we present the econometric methodology used in the paper. Modeling economic daily time series poses several and difficult challenges due to the coexistence of multiple seasonal components linked to various frequencies, the complex structure of the calendar,^2^ the strength of its irregular component and its sensitivity to exogenous factors (e.g., outliers) that distort its usual behavior (Ladiray et al. 2018).

We use the structural model of unobserved components proposed by De Livera et al. (2011) to perform modeling and seasonal adjustment of the Spanish daily sales data. The model, called TBATS (acronym for Trigonometric seasonality, Box–Cox transformation, ARMA innovations, Trend and Seasonality), complemented with a suitable dynamic-regression preprocessing, provides a flexible although parsimonious way to handle the complex nature of daily time series. The econometric methodology has two steps:

- Preprocessing (linearization). In this step, we apply an intervention analysis by means of exogenous deterministic variables designed to control for the presence of outliers and specific calendar effects that, due to their moving nature, do not fit well into the structural representation considered by TBATS (Hillmer and Bell 1983; Hillmer et al. 1983). This intervention analysis is a preprocessing step of the observed series that renders it suitable for TBATS.
- Structural decomposition using TBATS. The preprocessed time series is decomposed into trend-cycle, seasonality and irregularity. As we will expose below, the seasonal component has a complex nature due the coexistence of multiple seasonal patterns, some of them with fractional periodicities.

Let us now explain both steps with some detail.

### Preprocessing (linearization)

The rank-mean analysis of the time series, as well as its wide range of variation, suggests the convenience of using a preliminary logarithmic transformation. In the same vein, the estimation of the λ parameter of the Box–Cox transformation performed by TBATS is very close to zero,^3^ providing additional support for the log-transformation.

The analysis of the residuals both from the structural TBATS model and from a reduced-form model (Cuevas et al., 2019) confirms the need to control for some effects linked to: (i) bank holidays, (ii) the inexact periodicity of the monthly seasonal component and (iii) its interaction with the weekly seasonal component.

These effects are represented by deterministic variables, and their impact on the observed time series is estimated using a regression model that includes a linear trend and a multiple seasonal component affine to the one used in the trigonometric seasonal representation used by TBATS. The regression model is given as follows:

1 $$ z_{t} = \beta x_{t} + \alpha_{0} + \alpha_{1} t + \mathop \sum \limits_{i = 1}^{3} \mathop \sum \limits_{j = 1}^{{k_{i} }} \left[ {\gamma_{j} \sin \left( {\frac{2j\pi t}{{m_{i} }}} \right) + \varphi_{j} \cos \left( {\frac{2j\pi t}{{m_{i} }}} \right)} \right] + e_{t} $$

Being:

- *z*_*t*_: (log-transformed) observed variable.
- *x*_*t*_: *m* deterministic (dummy) variables linked to the bank holidays and to the inexact periodicity of the monthly seasonality.
- *m*_*i*_: periodicity of the *i*-th seasonal component. Based on a preliminary analysis (Cuevas et al. 2019), we have considered three components (weekly, monthly and yearly) whose periodicities,^4^ expressed in days, are 7, 30.4375 and 360.25.
- *k*_*i*_: number of harmonics of each seasonal component.
- *e*_*t*_: Gaussian error term.

It is interesting to note that the regression model (1) can be considered as a one-equation approximation to the complete structural TBATS model that will be presented below, especially due its similar treatment of the (multiple) seasonality. This similitude enhances the complementarity of steps 1 and 2.

The number of harmonics, *k*_*i*_, associated with each seasonal component (weekly, monthly and annual) can be determined by means of a preliminary estimate of the TBATS model applied to the original time series.

### Structural (TBATS) decomposition

The TBATS approach is based on the representation of the unobserved components (trend, seasonality, irregularity) by means of explicit dynamic models (Harvey 1989).^5^

Following the structural approach, the model incorporates a parsimonious but rather general representation of the trend. It also includes an explicit model for the irregular component that acts as a sort of “safety valve,” accommodating elements that, for whatever reason, did not find a proper fit within the basic systematic components (trend and seasonality). In this way, the plain representation of these two components does not compromise neither the fit of the model to the sample nor its forecasting performance.

The TBATS model assumes that the (possibly Box–Cox transformed) observed series (*z*_*t*_) results from the aggregation of three unobserved components: trend (*p*_*t*_), seasonality (*s*_*t*_) and a stationary innovation (*u*_*t*_). This innovation plays an additional role as the stochastic input for the other two components. In this way, both the trend and the seasonality depend on a single shock that, properly scaled and filtered, generates them. In general, we assume that *u*_*t*_ evolves according to a stationary and invertible autoregressive and moving average (ARMA) model:

2 $$ \left( {1 - \phi_{1} B - \ldots - \phi_{p} B^{p} } \right)u_{t} = \left( {1 - \theta_{1} B - \ldots - \theta_{q} B^{q} } \right)e_{t} $$

The ultimate shock *e*_*t*_ is a Gaussian white noise:

3 $$ e_{t} \sim iid N\left( {0, v} \right) $$

An interesting feature of TBATS is that it can handle complex seasonal patterns, comprising both multiple periodicities (weekly, monthly and yearly) and fractional periodicities (e.g., 30.4375 days for monthly seasonality or 365.25 for annual seasonality).

This complex seasonal pattern is one of the most important differences between a daily time series and its monthly/quarterly counterpart. In the latter case, the seasonal component is unique and of integer periodicity (12 months and 4 quarters, respectively). Of course, this additional complexity requires an additional layer of specific modeling.

Assuming that there are *I* seasonal components of different periodicity, total seasonality is the sum of all of them:

4 $$ S_{t} = \mathop \sum \limits_{i = 1}^{I} S_{t}^{\left( i \right)} $$

Each seasonal subcomponent is linked to a basic frequency and with k of its harmonics, according to the following equation:

5 $$ w_{j}^{i} = \frac{2\pi j}{{m_{i} }} $$

Being:

- *w*_*i,j*_ is the frequency of the *j*-th harmonic linked to the *i*-th seasonal subcomponent.
- *m*_*i*_ is the periodicity, in time units, of the seasonal subcomponent (e.g., 7 days for the weekly seasonality).

In this way, the seasonality associated with each basic frequency is obtained by adding the signals associated with that basic frequency and its *k* harmonics:

6 $$ S_{t}^{\left( i \right)} = \mathop \sum \limits_{j = 1}^{{k_{i} }} S_{j,t}^{\left( i \right)} $$

These individual terms are determined according to a bivariate vector autoregressive (VAR) process that includes S and an auxiliary factor *Q*.^6^

7 $$ \left[ {\begin{array}{*{20}c} {S_{j,t} } \\ {Q_{j,t} } \\ \end{array} } \right] = \left[ {\begin{array}{*{20}c} {{\text{cos}}\left( {w_{j} } \right)} & {{\text{sin}}\left( {w_{j} } \right)} \\ { - {\text{sin}}\left( {w_{j} } \right)} & {{\text{cos}}\left( {w_{j} } \right)} \\ \end{array} } \right]\left[ {\begin{array}{*{20}c} {S_{j,t - 1} } \\ {Q_{j,t - 1} } \\ \end{array} } \right] + \left[ {\begin{array}{*{20}c} {\gamma_{1} } \\ {\gamma_{2} } \\ \end{array} } \right]u_{t} $$

Equation (7) is a deterministic Fourier expansion centered on the frequency (5), stochastically perturbed by the common innovation of the system, *u*_*t*_. This innovation is scaled using the parameters *γ*_1_ and *γ*_2_. This representation stands out for its parsimony, since only four parameters are involved: two scale parameters and two initial conditions, regardless of the time scale of the seasonality, which can be very large: greater than 28 and 360 periods in the monthly and annual case, respectively.

In this way, for each seasonal subcomponent (e.g., weekly), *k* first-order VAR representations are defined, as many as harmonics are needed to represent it.

The magnitude of the scale parameters determines the proximity to a deterministic behavior of the seasonal subcomponent. At the limit, if both are zero, the component is completely deterministic.

Finally, the trend *p*_*t*_ is a random walk, *I*(1), with a first-order autoregressive, AR(1), drift:

8 $$ p_{t} = p_{t - q} + \phi g_{t - 1} + \alpha u_{t} $$

Being:

- *g*_*t*_: drift.
- *Φ*: damping parameter that controls for the impact of the drift on the trend. In general, 0 ≤ *Φ* ≤ 1.
- *α*: scale parameter that modules the impact of the innovation on the trend. As a rule, *α* ≥ 0.

The next equation defines the drift:

9 $$ g_{t} = \left( {1 - \phi } \right)b + \phi g_{t - 1} + \beta u_{t} $$

Being:

- *b*: location parameter that represents the steady state of the drift, provided that *Φ* < 1.
- *β*: scale parameter that modules the impact of the common innovation on the drift. In general, *β* ≥ 0.

Equations (8) and (9) provide a parsimonious yet flexible representation for the trend. In this way, depending on *Φ,* we can get an *I*(2) or an *I*(1) trend. If *Φ* = 1, we obtain an IMA(2,1) trend. In addition, the scale parameters determine the closeness to a deterministic behavior. As a special case, if 0 < *Φ* < 1 and *β* = 0, we get a random walk with a constant drift. If, in addition, *α* = 0, the trend becomes completely deterministic.

The TBATS procedure sets the model in state space form, computes its likelihood and maximizes it using the model parameters as instruments. It also determines the most appropriate Box–Cox transformation and, once applied, the proper number of harmonics for the seasonal subcomponents, starting with *j* = 1. In all the cases, the different combinations are ranked according to the Akaike information criterion (AIC) and the one that minimizes AIC is chosen.

Finally, TBATS performs a search for the most adequate ARMA(*p*,*q*) model for the innovation, starting with a white noise (*p* = *q* = 0). If the innovation fails to be considered as a white noise, a search along *p* and *q* is implemented, selecting the combination that minimizes the AIC.

## Empirical results

We turn now to the empirical results derived from the application of the methodology presented in the previous section, following its two-step scheme.

### Modeling aggregate daily sales

The first step consists of estimating Eq. (1). Let us now describe the exogenous variables considered in *x*_*t*_. The bank holidays variable is based on the official working calendar, including national and regional holidays. In this way, we get 20 daily time series (1 national, 17 regional and 2 for the autonomous cities). We have built a single regression variable by combining the 20 time series according to its weight on the distribution of interior sales as reported by the 56 offices of the Tax Agency (Cuevas et al. 2019).^7^ The role of this variable is improved if we restrict it to be binary, setting 2/3 as the threshold.

The effects linked to the deterministic part of the monthly seasonality are collected using three binary variables that separately consider whether the day is the beginning of the month, the 15th day, or the end of the month, adopting the value 1 in this case and 0 in the rest. These three effects interact with the weekly seasonality, for which three additional binary variables are considered that adopt the value of 1 if, in addition to being the beginning of the month, the 15th day or the end of the month, the day is also a weekend (Saturday or Sunday). Table 1 shows the results of the estimation of the deterministic effects, by means of the regression model (1).

**Table 1:** Estimation of deterministic effects

	Holiday	Monthly component					
		Basic effect			Interaction with the weekends		
		End of month	Beginning of month	15th day	End of month	Beginning of month	15th day
*β*	− 1.40	1.67	0.50	0.40	1.24	0.65	0.67
*t*(*β*)	− 34.00	31.44	9.17	8.11	16.30	8.63	8.84

The number of harmonics associated with each seasonal component (weekly, monthly and annual) is 3, 9 and 5, respectively. These values are derived from the preliminary estimate of a TBATS model applied to the original time series

It is worth noting the strength of the holiday effect, as well as the impact of the end of the month effect, in itself and in its interaction with the weekly seasonality. The beginning of the month effect and day-15th effect, although significant, are of a lesser magnitude.

The daily time series, corrected from the deterministic effects quantified in the previous table, is decomposed by means of the TBATS model. The next table presents the estimation of its parameters.

The estimated parameters indicate that all the seasonal components are quite stable, especially the one related to the weekly seasonality. This stability does not prevent the estimated components from being relatively complex, given the high number of harmonics required to represent them.

The estimated trend is a random walk whose specific innovation is much less volatile than the corresponding to the common innovation u_t_.

**Fig. 3:**
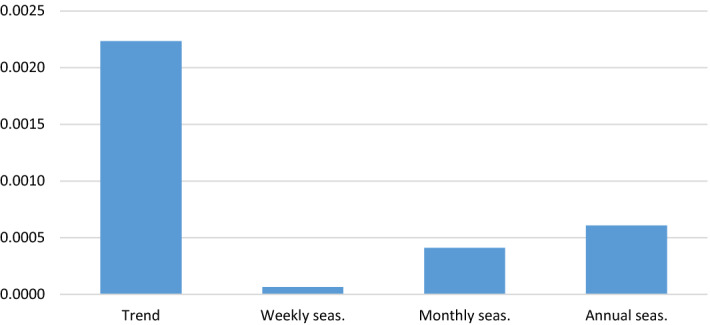
Transmission of an innovation to the components. *Note* The units are fractions of the standard deviation of the innovation

From the previous table, we can ascertain that the common innovations u_t_ that affect the series are mainly reflected in its trend and its annual seasonality. Monthly seasonality and, above all, weekly seasonality are relatively immune to the shocks. This ranking is an alternative way to quantify proximity to a deterministic scheme and is shown in Fig. 3.

**Fig. 4:**
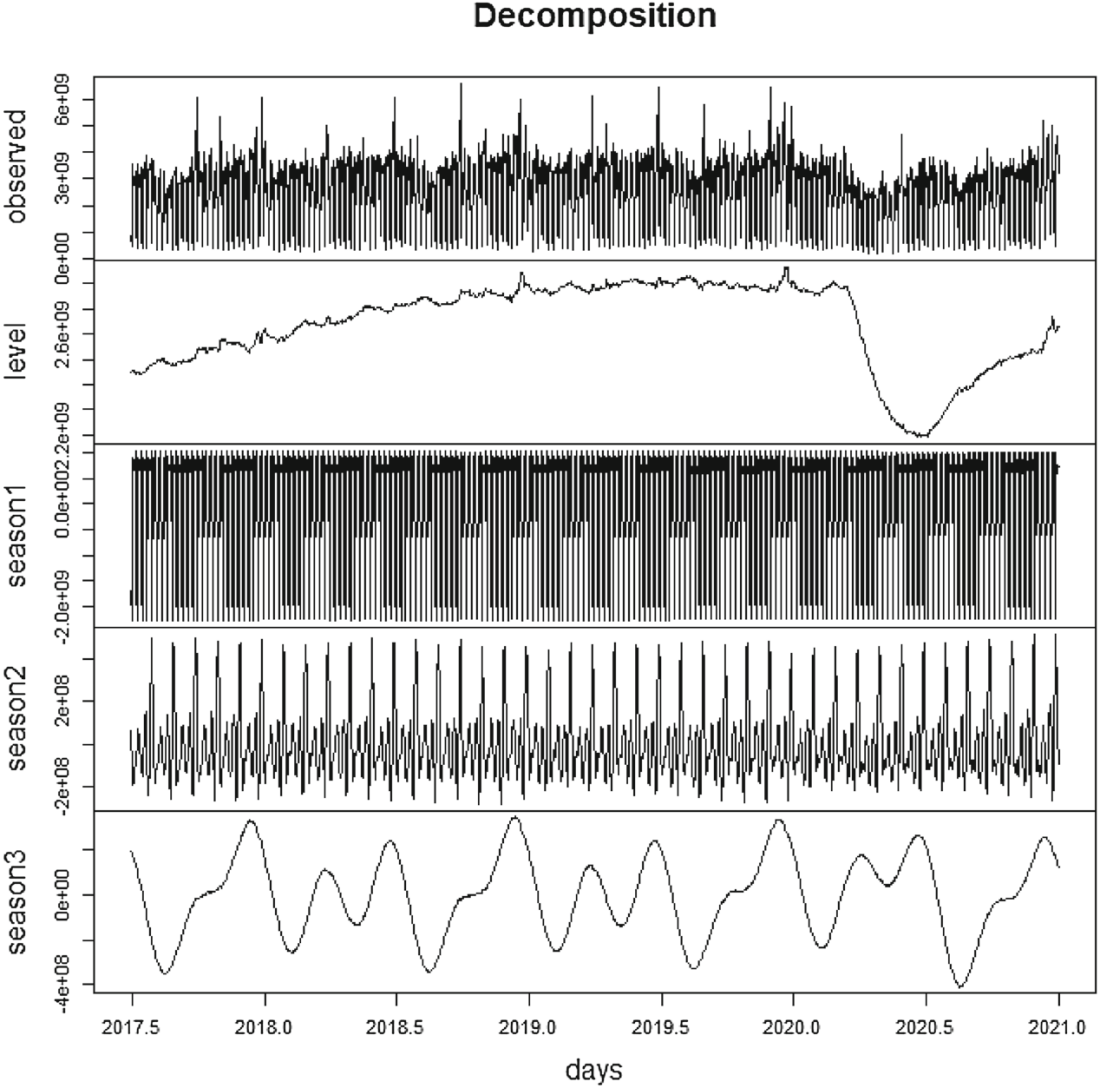
Stochastic decomposition of the daily SII sales. *Note* level refers to trend, season1 refers to weekly seasonality, season2 refers to monthly seasonality and season3 refers to annual seasonality

The TBATS model, whose parameters are shown in Table 2, allows the estimation of the unobserved components that underlie the observed series by means of the Kalman filter. These components are presented in Fig. 4.

**Table.2:** Estimation of TBATS parameters

Seasonality								
Weekly			Monthly			Annual		
< *m*, *k* >	*ϒ*_1_	*ϒ*_2_	< *m*, *k* >	*ϒ*_1_	*ϒ*_2_	< *m*, *k* >	*ϒ*_1_	*ϒ*_2_
< 7, 3 >	− 0.0003	0.0020	< 30.4375, 7 >	− 0.0022	0.0014	< 365.25, 6 >	− 0.0033	0.0010
Trend			Innovation: ARMA(*p*,*q*)					
*Φ*	*Α*	*β*	*p*	*q*	*σ*	Likelihood	AIC	*n*
0	0.012	0	0	0	0.4129	3473.39	3553.39	983

< *m*, *k* > denote the periodicity and the number of harmonics for each seasonal component, respectively

**Fig. 5:**
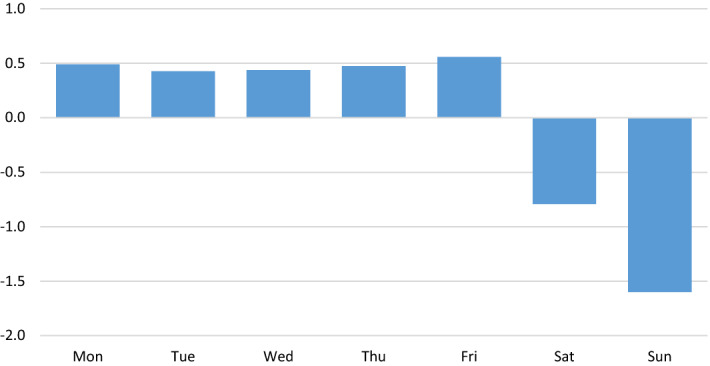
Mean profile of the weekly seasonality

The information provided by this decomposition makes it possible to infer, in the first place, the weekly seasonal profile, the most stable component of all. As can be seen in the following graph, the weekly pattern of daily sales shows a strong contrast between the weekend, especially Sunday, and the rest of the week (Fig. 5).

**Fig. 6:**
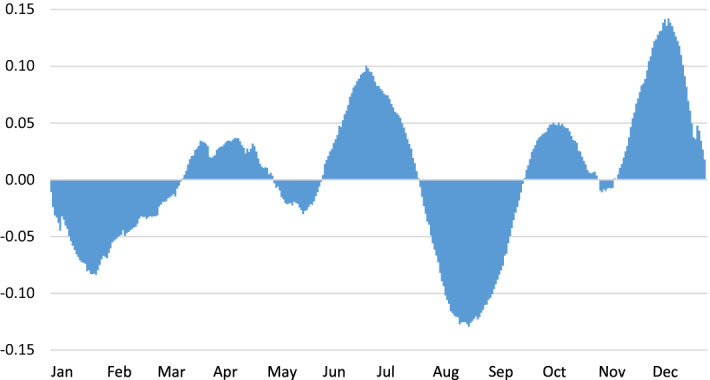
Mean profile of the annual seasonality

In the same vein, we can check the mean profile of the annual seasonality. As can be seen in the next figure, its pattern is relatively complex, comprising several peaks and troughs. Among the former, the days that belong to December, June and July stand out. On the other hand, the trough days are registered in August, January and February (Fig. 6).

**Fig. 7:**
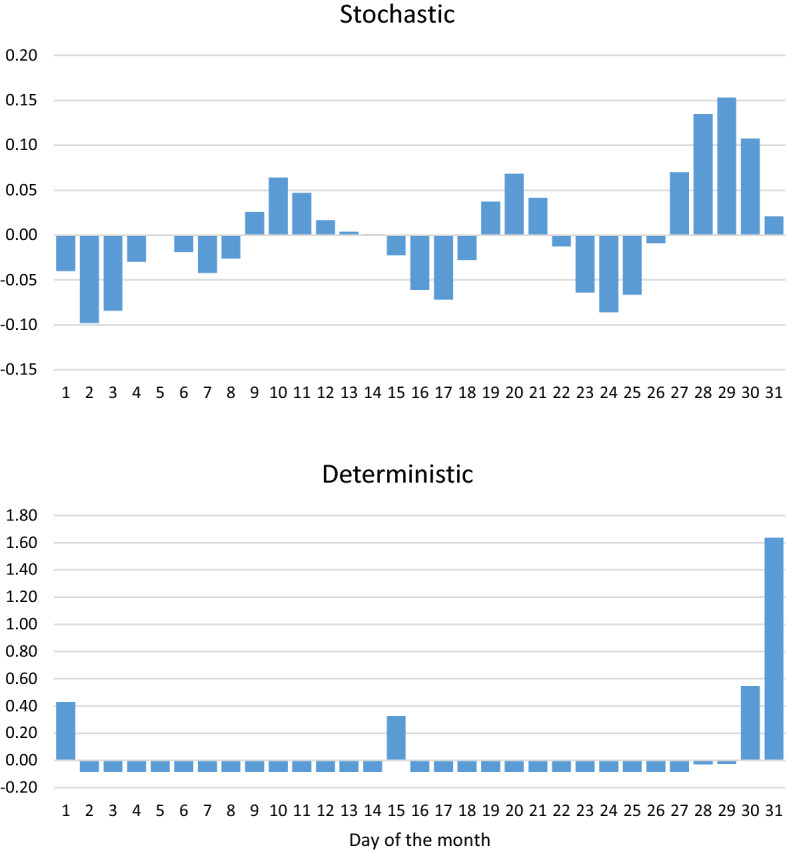
Mean profile of the monthly seasonality

To fully analyze the monthly seasonal profile, it is necessary to combine the stochastic component estimated by TBATS, as shown in Fig. 4, with the estimation of the deterministic effects of monthly periodicity, (beginning of the month, day 15th and end of the month effects), presented in Table 1. These effects, properly centered to average zero in the long term, are shown in the following graph (Fig. 7).

The main features of the monthly seasonality are, first and above all, the preeminence of its deterministic component, the important role of the end of the month effect and, finally, the lesser relevance of both the beginning of the month and the 15th day effects.

One additional feature of the TBATS model is that can be used to forecast the daily sales in order to have permanently filled the current month. Of course, the forecasts can be extended as much as required if, for whatever reasons, more distant forecasts are required. In Sect. 5, we present additional information about the predictive performance of the model.

### Results by activity breakdown

As mentioned before, the firms included in the Immediate Information Supply system (SII) are classified according to the activity declared by their own companies. Specifically, the activities are classified according to the breakdown of the Economic Activity Tax (IAE, *Impuesto de Actividad Económica*), which has a correspondence with the standard NACE-2009 classification (see “Annex 1” for details on its coverage).

In this way, the methodological approach previously described can be applied to all the branches of activity considered. For simplicity, we are going to show the most significant results for total sales and the main branches of activity. Additionally, we are going to focus in the recent recessive episode due to the COVID-19 health crisis, because the extraordinary and unusual nature of this shock deserves special attention, showing how it has affected the different economic activities.

**Fig. 8:**
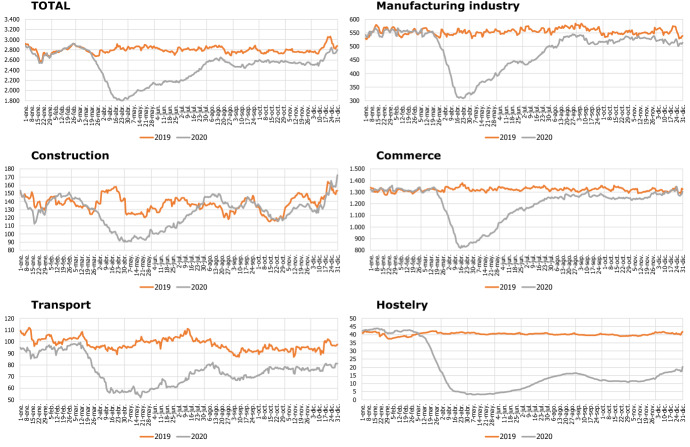
Final corrected series (MA28 levels)

**Fig. 9:**
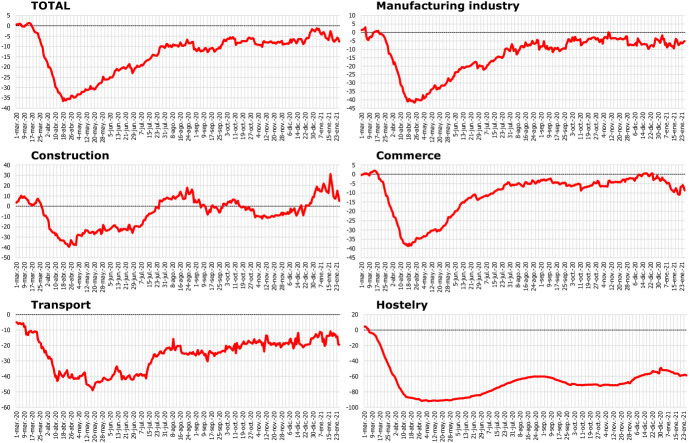
Final corrected series (MA28 levels): Year-on-year (YoY) growth rate

Figures 8 and 9 show, respectively, the final series corrected from deterministic effects and seasonal and calendar components, and their corresponding year-on-year rate growth. We have used a 28 days moving average filter to render the levels comparable with those of a monthly time series.

The adjustment carried out allows us to extract a clear signal, useful for the monitoring of sales in each sector on a real-time basis. Likewise, the decline in activity caused by the impact of the COVID-19 shock has been clearly uneven by activity branch. The greatest impact caused by the lockdown and related policies have had the largest impact on the hostelry and transport sectors, with falls that remain at 60% in the first case.

## Predictive performance

Daily forecasting is an important use of the TBATS model, in addition to seasonal adjustment. In this way, for example, we can complete the current month having an updated forecast that can be plugged in nowcasting models. Daily forecasts can also be used to assess and monitor the short-term situation. The exercise proposed in this section seeks to evaluate the forecasting accuracy of this model against other alternatives that generate daily projections.

The training set to be considered will be the SII series up to 2019/12/31, while 2020 would be used as the test set. It is necessary to emphasize that information is only available since mid-2017, so in order to have one-year forecast horizon and at least 20% of the sample for the test set (Hyndman and Athanasopoulos 2021), we cannot evaluate previous years. Therefore, TBATS forecasting accuracy is checked during an atypical period due to the effect of the COVID-19 shock.

**Fig. 10:**
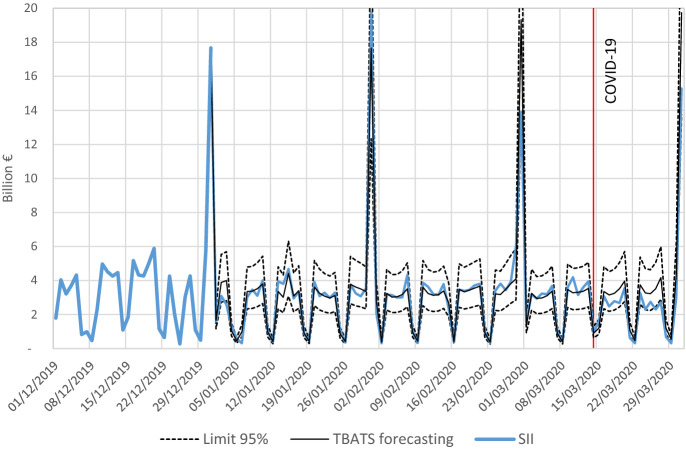
Out-of-sample forecasts from 2019/12/31 to 2020/03/31 versus real data

Figure 10 shows 2020 the first quarter out-of-sample forecast from 2019/12/31 versus real data. As can be seen, TBATS captures well the seasonality and returns a similar level to the actual data for the forecast horizon. It only shows a deviation from mid-March due to the COVID-19 shock.

In order to further refine the evaluation of the predictive performance, the forecasting accuracy evaluation is done through two exercises based on rolling forecast schemes with different time frames. Firstly, a rolling forecast exercise day by day until the month is completed, on the other hand, a daily forecast scheme month by month until the end of the quarter, which will be extended secondly. As alternative models, versus the TBATS model as benchmark, the following methods are proposed:

1. RW: random walk.
2. STLM: STL (Seasonal and Trend decomposition using LOESS^8^) method.
3. ARIMA: ARIMA model obtained from AIC minimization.^9^
4. MRL: linear time series with trend and multiple seasonality.

These four methods cover various projection approaches: simple (RW), nonparametric (STLM), parametric in reduced form (ARIMA), and parametric based on an essentially deterministic regression model (MRL). Moreover, all the models have been constructed with the series in logarithms and corrected from the COVID-19 shock with dummy variables.

Finally, as a measure of predictive precision, the comparison between models is based on the root of the mean square error (RMSE). Besides, the second exercise completes the analysis using the Diebold and Mariano test for the null hypothesis that the two methods have the same forecast accuracy using the modified version proposed by Harvey et al. (1997).

**Fig. 11:**
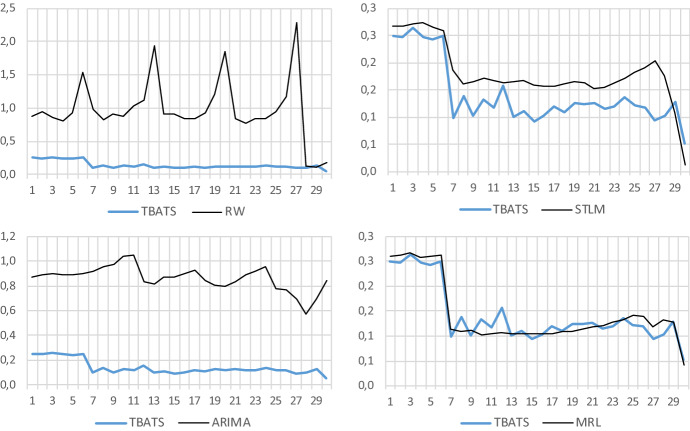
RMSE daily forecast rolling scheme

Table 3 and Fig. 11 show the RMSE of the daily forecast until the end of January 2020. TBATS clearly improves the results of the RW and the ARIMA model. The MRL improves it in specific moments, while the STLM presents a lower RMSE only when the size of the forecast horizon is small, that is, when there are a few days left to finish the month.

**Table 3:** Rolling forecast scheme day by day until the end of the month

Forecast horizon	Model				
	TBATS	RW	STLM	ARIMA	MRL
*RMSE of test sets*					
01/01/2020–31/01/2020	0.25	0.87	0.27	0.87	0.26
02/01/2020–31/01/2020	0.25	0.94	0.27	0.89	0.26
03/01/2020–31/01/2020	0.26	0.86	0.27	0.90	0.27
04/01/2020–31/01/2020	0.25	0.81	0.27	0.89	0.26
05/01/2020–31/01/2020	0.24	0.92	0.26	0.89	0.26
06/01/2020–31/01/2020	0.25	1.53	0.26	0.90	0.26
07/01/2020–31/01/2020	0.10	0.98	0.19	0.92	0.11
08/01/2020–31/01/2020	0.14	0.83	0.16	0.96	0.11
09/01/2020–31/01/2020	0.10	0.91	0.17	0.97	0.11
10/01/2020–31/01/2020	0.13	0.88	0.17	1.04	0.10
11/01/2020–31/01/2020	0.12	1.03	0.17	1.05	0.10
12/01/2020–31/01/2020	0.16	1.12	0.16	0.83	0.11
13/01/2020–31/01/2020	0.10	1.93	0.17	0.82	0.10
14/01/2020–31/01/2020	0.11	0.91	0.17	0.87	0.10
15/01/2020–31/01/2020	0.09	0.91	0.16	0.88	0.10
16/01/2020–31/01/2020	0.10	0.85	0.16	0.90	0.11
17/01/2020–31/01/2020	0.12	0.84	0.16	0.93	0.10
18/01/2020–31/01/2020	0.11	0.93	0.16	0.84	0.11
19/01/2020–31/01/2020	0.13	1.21	0.16	0.80	0.11
20/01/2020–31/01/2020	0.12	1.84	0.16	0.79	0.11
21/01/2020–31/01/2020	0.13	0.84	0.15	0.83	0.12
22/01/2020–31/01/2020	0.12	0.77	0.15	0.89	0.12
23/01/2020–31/01/2020	0.12	0.84	0.16	0.92	0.13
24/01/2020–31/01/2020	0.14	0.85	0.17	0.96	0.13
25/01/2020–31/01/2020	0.12	0.95	0.18	0.78	0.14
26/01/2020–31/01/2020	0.12	1.17	0.19	0.76	0.14
27/01/2020–31/01/2020	0.09	2.29	0.20	0.69	0.12
28/01/2020–31/01/2020	0.10	0.13	0.18	0.57	0.13
29/01/2020–31/01/2020	0.13	0.10	0.11	0.70	0.13
30/01/2020–31/01/2020	0.05	0.17	0.01	0.84	0.04

**Fig. 12:**
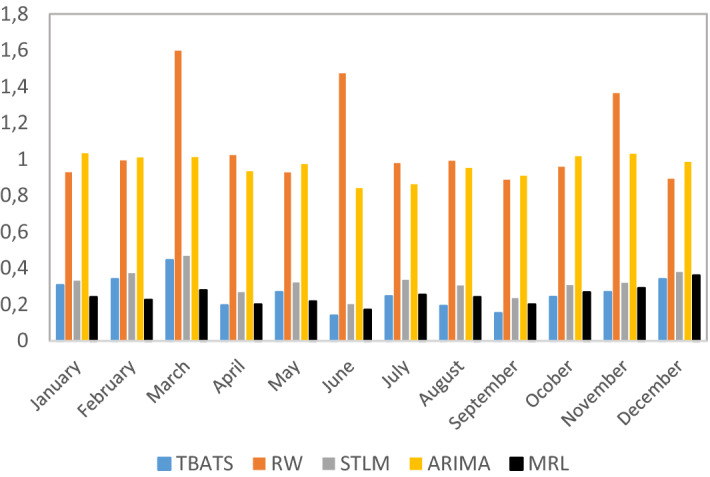
RMSE daily forecasting rolling scheme month by month

The results of the second exercise are collected in Table 4 and plotted in Fig. 12. As in the daily case, the three models with the best accuracy are TBATS together with the MRL and the STLM. However, MRL improves TBATS in the first quarter of the year, coinciding with the beginning of the pandemic. In the second quarter, TBATS beats the rest of the models in June. Finally, in the remaining months from the third quarter, TBATS registers a lower RMSE than the proposed alternatives.

**Table 4:** Rolling forecast scheme month by month until the end of the quarter

Forecast horizon	Model				
	TBATS	RW	STLM	ARIMA	MRL
*RMSE of test sets*					
*Q1*					
2020/01/01–2020/03/31	0.31	0.93	0.33	1.03	0.24
2020/02/01–2020/03/31	0.34	0.99	0.37	1.01	0.23
2020/03/01–2020/03/31	0.45	1.6	0.47	1.01	0.28
*Q2*					
2020/04/01–2020/06/30	0.20	1.02	0.27	0.93	0.20
2020/05/01–2020/06/30	0.27	0.93	0.32	0.97	0.22
2020/06/01–2020/06/30	0.14	1.47	0.2	0.84	0.17
*Q3*					
2020/07/01–2020/09/30	0.25	0.98	0.34	0.86	0.26
2020/08/01–2020/09/30	0.19	0.99	0.3	0.95	0.24
2020/09/01–2020/09/30	0.15	0.89	0.24	0.91	0.20
*Q4*					
2020/10/01–2020/12/31	0.24	0.96	0.31	1.02	0.27
2020/11/01–2020/12/31	0.27	1.36	0.32	1.03	0.29
2020/12/01–2020/12/31	0.34	0.89	0.38	0.98	0.36

Complementing the analysis, the results of the Diebold and Mariano test month by month through the *p* values are represented in Table 5. TBATS is the benchmark model against the proposed alternatives. It can be seen how the RW and the ARIMA models show different accuracy from the benchmark in all periods of the test sample. In the MRL case, there is a major rejection of the null hypothesis in the second semester once the model assimilates the impact of the pandemic. In the case of the STLM, the analysis of the results month by month does not provide a clear picture.

**Table 5:** Diebold-Mariano test

Forecast horizon	Model			
	RW	STLM	ARIMA	MRL
*Q1*				
2020/01/01–2020/01/31	0.01***	0.22	0.00***	0.49
2020/02/01–2020/02/29	0.00***	0.03**	0.00***	0.95
2020/03/01–2020/03/31	0.00***	0.71	0.00***	0.85
*Q2*				
2020/04/01–2020/04/30	0.00***	0.10*	0.00***	0.69
2020/05/01–2020/05/31	0.00***	0.10*	0.00***	0.51
2020/06/01–2020/06/30	0.00***	0.01***	0.00***	0.51
*Q3*				
2020/07/01–2020/07/31	0.00***	0.07*	0.00***	0.02**
2020/08/01–2020/08/31	0.00***	0.29	0.00***	0.02**
2020/09/01–2020/09/30	0.00***	0.16	0.00***	0.03**
*Q4*				
2020/10/01–2020/10/31	0.00***	0.22	0.01***	0.08*
2020/11/01–2020/11/30	0.00***	0.28	0.00***	0.20
2020/12/01–2020/12/31	0.02**	0.06*	0.00***	0.15

1. Diebold-Mariano test (the Harvey et al.^1997^, version) for the null hypothesis of the equal forecast accuracy of two forecast methods. The number represents the *p* value

*Rejection of the null hypothesis at the 10% level of significance

**Rejection of the null hypothesis at the 5% level of significance

***Rejection of the null hypothesis at the 1% level of significance

Summarizing, TBATS model provides a lower RMSE than the proposed alternatives in most of the prediction intervals considered. Besides, the Diebold and Mariano test rejects in most of the cases the null hypothesis of same forecast accuracy. The deviations from the forecast on real data occur from the COVID-19 outbreak. However, the reliability of the method is comparable to other models in this atypical period.

## Conclusions

The use of structural, unobserved components time series model has been successful both to extract non-seasonal information and to forecast the daily data.

From a purely statistical view, the employ of tax data stands out as an inexpensive and reliable way to use the information routinely provided by the economic agents when complying with their tax duties, replacing the use of costly sample surveys or qualitative information derived from data sources whose representativeness cannot be properly assessed. Of course, part of this reliability comes from the positive reinforcement due to penalties and fines if compliance is not perfect. Few data sources are backed by this reinforcement schedule.

For future work, we plan to compare TBATS with both STLM (Ollech 2018) and with Prophet (Taylor and Letham 2018) in the realm of seasonal adjustment, in order to ascertain the performance of model-based methods (TBATS) versus nonparametric filtering methods (STLM) and Bayesian regression methods (Prophet).

## Annex 1: Breakdown of sales

The branches of activity that can be considered, with their corresponding coverage in 2019, comprise the following sections of the NACE:

**Table:**

	Number of companies		Domestic sales (mill. €)		Percentage of domestic sales of SII companies with respect to the total
	SII	Total	SII	Total	
TOTAL DOMESTIC SALES	57,855	37,96,021	12,13,602	17,30,463	70.1
C. Manufacturing industry	11,499	1,75,039	2,45,057	3,07,734	79.6
C10. Food industry	2605	25,819	64,929	75,588	85.9
C11 + C12. Manufacture of beverages and tobacco	703	5775	12,563	14,754	85.2
C13 + C14 + C15. Textile industry, garment manufacturing and leather and footwear industry	935	22,910	6545	11,254	58.2
C17 + C18. Paper industry; graphic arts	559	15,844	10,537	15,256	69.1
C20. Chemical industry	810	3866	21,475	24,215	88.7
C21. Manufacture of pharmaceutical products	233	473	12,225	12,867	95.0
C22 + C23. Manufacture of rubber and plastic products and other non-metallic mineral products	1256	11,866	19,336	25,477	75.9
C24 + C25. Metallurgy; manufacture of iron, steel, ferro-alloy and metal products, except machinery and equipment	1686	33,920	23,679	36,751	64.4
C26 + C27. Manufacture of computer, electronic and optical products; manufacture of electrical material and equipment	457	4158	7655	9147	83.7
C29. Manufacture of motor vehicles, trailers and semi-trailers	370	1635	30,628	33,859	90.5
C16 + C31. Wood and cork industry; furniture manufacturing	604	22,114	5086	10,533	48.3
C28 + C30 + C33. Manufacture of machinery and equipment; manufacture of other transport equipment; repair and installation of machinery and equipment	1063	19,290	10,806	17,202	62.8
C19 + C32. Coke and refined petroleum products: other manufacturing industries	218	7369	19,595	20,831	94.1
D. Supply of electricity, gas, steam and air conditioning	760	29,695	36,647	49,318	74.3
F. Construction	6959	4,09,901	61,406	1,23,140	49.9
F41. Building construction	4114	2,35,355	28,841	68,620	42.0
F42. Civil Engineering	679	12,989	9603	14,396	66.7
F43. Specialized construction activities	2166	1,61,557	22,963	40,124	57.2
G. Wholesale and retail trade; motor vehicle and motorcycle repair	19,964	4,96,720	5,47,723	6,84,794	80.0
G45. Sale and repair of motor vehicles and motorcycles	2133	76,504	70,328	85,865	81.9
G46. Wholesale trade and trade intermediaries, except of motor vehicles and motorcycles	15,140	2,12,881	3,41,390	4,05,586	84.2
G47. Retail trade, except of motor vehicles and motorcycles	2691	2,07,335	1,36,005	1,93,343	70.3
G471. Retail trade in non-specialized stores	378	24,833	80,038	91,693	87.3
G473. Retail sale of automotive fuel in specialized stores	486	4871	11,772	18,618	63.2
G472, G474 to G479. Rest of retail trade	1827	1,77,631	44,195	83,032	53.2
H. Transportation and storage	2975	1,81,033	51,556	80,024	64.4
I. Hostelry	1456	2,69,340	18,344	65,287	28.1
I55. Accommodation Services	907	33,369	11,049	22,640	48.8
I56. Food and beverage services	549	2,35,971	7295	42,647	17.1
J. Information and communications	1642	69,112	57,953	70,739	81.9
M + N. Professional and administrative activities	5600	6,47,369	1,12,607	1,75,550	64.1
Z. Rest of activities	7000	15,17,812	82,308	1,73,877	47.3

## Footnotes

1 A group of companies is considered when it is formed by a parent company and its subsidiaries. They must be firmly bound to one another.

2 The most notable effects are different length and composition of the months, moving seasonality (e.g., Easter), leap years and a time-varying calendar of working days that interacts with the composition of the months.

3 The exact value is 0.0039.

4 The periodicity of the monthly seasonal component takes into account both the different length of the months and the leap years. The fractional periodicity of the annual seasonality is only due to the presence of leap years.

5 The alternative approach, based on reduced-form models, offers greater flexibility to include exogenous variables but does not provide an estimate of the underlying components (Espasa et al. 1996; Liu 2005). Although less complete, we have also used this approach as a cross-check (Cuevas et al. 2019).

6 Q is a shifted version of S that helps to complete the state space representation (6).

7 An alternative estimate, using weights from the Spanish Regional Accounts, yields similar results.

8 LOESS means Locally Estimated Scatterplot Smoothing (see Cleveland et al. 1990).

9 The model selected for the initial training sample (from 2017/07/01 to 2019/12/31) was a ARIMA(4,0,3)(0,1,0)[365] with drift and [365] is the seasonal frequency.
